# Stress Ulcer Prophylaxis in Critical Care: Evidence, Risk Stratification, and Clinical Implications

**DOI:** 10.7759/cureus.106746

**Published:** 2026-04-09

**Authors:** Priscila Sole

**Affiliations:** 1 Medicine, Centro Universitário Lusíada, Santos, BRA; 2 Critical Care Medicine, The University of Edinburgh, Edinburgh, GBR

**Keywords:** critical care, critically ill patients, enteral nutrition, gastrointestinal bleeding, intensive care unit, proton pump inhibitors (ppis), risk stratification, stress-related mucosal damage, stress ulcer prophylaxis, upper gastrointestinal (ugi) bleeding

## Abstract

Stress-related mucosal damage (SRMD) is a common complication in critical care medicine. The necessity of stress ulcer prophylaxis (SUP) is debated due to advancements in supportive care and challenges in accurately stratifying bleeding risk.

This narrative review evaluates contemporary evidence on stress ulcer prophylaxis in critically ill patients, with emphasis on bleeding risk stratification and implications for clinical practice.

To construct this narrative review, we searched Embase and PubMed for randomized controlled trials (RCTs) evaluating SUP in adult intensive care unit (ICU) populations. The Population, Intervention, Comparison, and Outcome (PICO) framework was used to guide the search through March 2026. Major trials, relevant subgroup analyses, meta-analyses, and contemporary international clinical practice guidelines were reviewed to contextualize trial-level findings.

Large randomized controlled trials demonstrated that acid-suppressive therapy reduces the incidence of clinically significant gastrointestinal bleeding (GIB), without demonstrating a mortality benefit. Inconsistent definitions of risk factors for GIB limit reproducibility and reduce the precision of pooled risk estimates. Meta-analyses confirmed a reduction in GIB and highlighted clinical heterogeneity. Current guidelines recommend stress ulcer prophylaxis for patients with high-risk presentations. However, these recommendations remain conditional due to the absence of a multivariable prediction model.

Despite current uncertainty regarding the effects of stress ulcer prophylaxis on gastrointestinal bleeding prevention and mortality, it is recommended to consider SUP in selected critically ill patients based on individual risk assessment. In parallel, clinicians should optimize supportive measures, including early enteral nutrition, hemodynamic stabilization, and discontinuation of prophylaxis once the patient is clinically stable. Further prospective research is needed to guide individualized prophylaxis and avoid unnecessary treatment in low-risk populations.

## Introduction and background

Stress-related mucosal damage (SRMD) is a well-recognized consequence of critical illness. Patients admitted to the intensive care unit (ICU) are particularly susceptible to gastrointestinal (GI) mucosal injury due to hemodynamic instability, systemic inflammatory responses, impaired splanchnic perfusion, and varying degrees of multi-organ dysfunction. Endoscopic studies demonstrated that gastric mucosal abnormalities can be detected in up to 90% of individuals within the first 72 hours of ICU admission [1,2].

Despite the high frequency of endoscopic mucosal changes, the incidence of clinically significant gastrointestinal bleeding (drop in hemoglobin of >20 g/L) is considerably lower. Contemporary data indicate that clinically important bleeding occurs in approximately 2%-5% of critically ill adults. This discrepancy likely reflects improvements in modern critical care. Advances such as timely fluid resuscitation, more effective hemodynamic stabilization, and the early initiation of enteral nutrition have contributed to preserving mucosal integrity and reducing the progression from superficial erosions to clinically relevant hemorrhage [1-7].

However, when clinically significant gastrointestinal bleeding (GIB) does occur, it is associated with important complications, including increased transfusion requirements, longer ICU stays, and worse overall clinical outcomes. Given these potential events, stress ulcer prophylaxis (SUP) became routine practice in intensive care units [3,8].

At the same time, concerns regarding the adverse effects of prolonged acid suppression emerged. Although the association between proton pump inhibitors (PPIs) and infectious complications, such as ventilation-associated pneumonia or *Clostridium difficile* infection, has not been consistently demonstrated across studies, the possibility of unintended harm has contributed to growing debate. This uncertainty has led clinicians to question the appropriateness of administering prophylaxis indiscriminately to all critically ill patients [5,9-11].

Contemporary practice has shifted toward a risk-stratified approach. International guidelines now recommend SUP primarily for critically ill patients with established risk factors for clinically important bleeding. However, these recommendations are conditional, reflecting uncertainty in how bleeding risk is defined and operationalized in modern ICU populations [12,13].

This review aims to synthesize current evidence on stress ulcer prophylaxis in critical care by examining how bleeding risk has been defined across contemporary studies. By comparing key randomized trials, subgroup analyses, meta-analyses, and current guideline recommendations, this review seeks to clarify the existing evidence and explore opportunities to improve risk stratification, inform clinical decision-making, and guide future research.

## Review

Methodology

This narrative review was structured around a predefined Population, Intervention, Comparison, and Outcome (PICO) framework to guide evidence synthesis.

A structured literature search was conducted across the databases Embase (Ovid, Embase Classic + Embase) and MEDLINE through March 2026. Only studies published in English were included, using indexed headings, subheadings, and relevant keywords related to stress ulcer prophylaxis and critical illness.

The search strategy included combinations of the following keywords: "critically ill patient”, “intensive care unit”, “stress ulcer prophylaxis”, “upper gastrointestinal bleeding”, and “gastrointestinal hemorrhage”, combined using Boolean operators (AND, OR).

After initial retrieval, results were limited to randomized controlled trials (RCTs) to prioritize higher-level comparative evidence. The final search included 10 randomized controlled trials that met the inclusion criteria.

**Figure 1:**
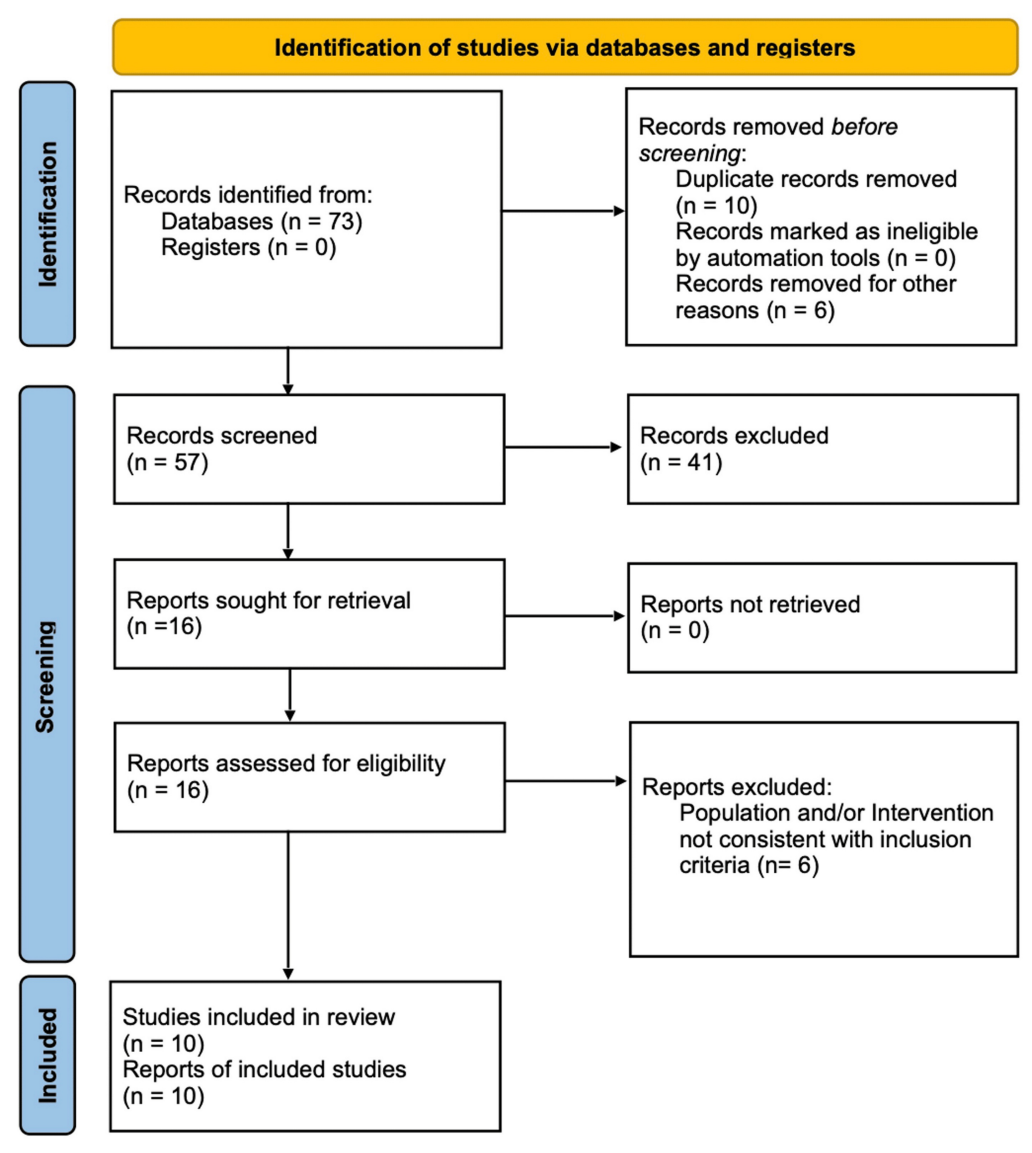
PRISMA 2020 flow diagram illustrating the study selection PRISMA: Preferred Reporting Items for Systematic Reviews and Meta-Analyses

A Preferred Reporting Items for Systematic Reviews and Meta-Analyses (PRISMA) flow diagram of included studies is shown in Figure 1.

The population included adult critically ill patients (≥18 years old) admitted to intensive care units. The interventions used were stress ulcer prophylaxis strategies, including pharmacologic therapy (proton pump inhibitors or histamine-2 receptor antagonists) and enteral nutrition-based approaches. Comparators included placebo, no prophylaxis, alternative pharmacologic agents, or enteral nutrition alone. The outcomes were clinically important gastrointestinal bleeding, all-cause mortality, and infectious complications.

Studies were included if they evaluated stress ulcer prophylaxis strategies in adult critically ill patients and reported at least one prespecified outcome of interest. Pediatric populations, case reports, protocols, and pilots were excluded.

Data were extracted in a structured format, including study design, population characteristics, intervention details, comparator groups, clinical outcomes, and considerations of internal and external validity. Dosing strategies varied across studies and were not standardized in this review, as the focus is on the overall effectiveness of stress ulcer prophylaxis and high-risk patients rather than dose-specific outcomes.

The primary search focused on randomized controlled trials. Additionally, meta-analyses, contemporary clinical guidelines, and textbook chapters were also reviewed to provide supplemental additional context.

Findings were synthesized methodically, with an emphasis on large multicenter RCTs and contemporary evidence to guide clinical practice.

All references were managed using EndNote (Clarivate, London, UK).

Pathophysiology

The pathophysiology underlying widespread superficial mucosal injury in stress-related mucosal damage (SRMD) is key to understanding the contemporary debate over routine prophylaxis and the identification of potential bleeding risk factors.

Stress-related mucosal damage arises early in the course of critical illness and results from systemic hemodynamic instability and compromised mucosal defense mechanisms. In contrast to chronic peptic ulcer disease, which is predominantly caused by acid exposure and *Helicobacter pylori* infection, SRMD is characterized by splanchnic hypoperfusion, reperfusion injury, and barrier dysfunction [1,2,11,14].

Splanchnic hypoperfusion is central to the pathogenesis of SRMD. Shock, systemic inflammation, and vasopressor therapy shift blood away from the gastrointestinal tract, leading to mucosal ischemia. Following hypoperfusion, gastrointestinal motility is decreased, which leads to prolonged exposure of the mucosa to gastric acid. Additionally, when reperfusion occurs, elevated levels of nitric oxide generate an inflammatory response. This scenario creates cellular damage and a tendency to ulceration. Therefore, even after apparent stabilization of systemic hemodynamics, persistent microvascular alterations may continue to cause epithelial disruption and superficial erosions [1,2,12,14].

Under normal physiological conditions, the gastric mucosa is protected by several coordinated defense mechanisms, including the mucus-bicarbonate barrier, epithelial integrity, prostaglandin-mediated protection, and adequate mucosal blood flow. Critical illness disrupts these protective systems, thereby increasing mucosal vulnerability to injury. As a result, endoscopic abnormalities are frequently observed in critically ill patients, with reported rates between 74% and 100% within the first 72 hours of intensive care unit admission [1,5,14].

Despite the high prevalence of gastrointestinal mucosal changes, the incidence of clinically significant bleeding has been markedly lower in modern ICU practice [5].

Although acid hypersecretion is not the main cause of mucosal lesions, gastric acid can exacerbate the injury because the mucosal defenses are compromised. Current studies support that perfusion abnormalities, rather than acid excess alone, are the principal factors underlying clinically significant bleeding [1,3].

Risk factors for clinically important gastrointestinal bleeding

Identification of patients at increased risk of clinically important gastrointestinal bleeding has been central to stress ulcer prophylaxis strategies. Cook et al. identified prolonged mechanical ventilation (>48 hours) and coagulopathy as independent predictors of clinically significant bleeding in critically ill adults [3].

Subsequent observational studies have demonstrated an association between bleeding risk and overall illness severity. Shock requiring vasopressors, chronic liver disease, and major abdominal surgery have been considered and described as high-risk conditions [2,5,15].

However, the absolute incidence of clinically important bleeding in patients without established risk factors appears to be low in modern ICU practice, often below 1% [3].

Additional medical conditions, as summarized in Table 1, include acute kidney injury (AKI), renal replacement therapy (RRT), and acute neurologic injury, which have been proposed as potentially higher-risk states due to hemodynamic instability, autonomic dysregulation, and impaired mucosal perfusion. Evidence-based data specifically evaluating these subgroups remains limited [6,7,16,17].

**Table 1:** Risk domains for clinically important gastrointestinal bleeding in clinical ill patients RCTs: randomized controlled trials, ICU: intensive care unit

Risk domain	Representative factors	Biological rationale	Evaluation in contemporary RCTs
Hemodynamic compromise	Shock, vasopressor requirement, prolonged mechanical ventilation	Splanchnic hypoperfusion and ischemia	Recognized as major risk factors and commonly incorporated into eligibility criteria in randomized trials
Coagulation abnormalities	Coagulopathy, thrombocytopenia, chronic liver disease	Impaired hemostasis	Frequently recognized as risk factors, not consistently evaluated in randomized trials
Organ dysfunction/systemic inflammatory states	Renal replacement therapy, sepsis, major trauma, severe burns	Systemic inflammation, platelet dysfunction, and microvascular injury contributing to mucosal damage	Mainly explored through subgroup analyses
Neurologic injury	Traumatic brain injury, subarachnoid hemorrhage, neurosurgical ICU patients	Autonomic dysregulation and gastric acid hypersecretion	Limited randomized evidence; primarily small single-center studies
Medication-related factors	Anticoagulation, antiplatelet therapy, corticosteroids	Pharmacologic impairment of clot formation and mucosal hemostasis	Rarely evaluated across randomized trials
Cumulative risk burden (≥2 domains)	Combined hemodynamic instability, coagulopathy, organ dysfunction, acute neurologic injury, medications	Potential synergistic amplification of bleeding risk in complex critical illness	Not directly evaluated in contemporary randomized trials

Secondary analyses of contemporary randomized trials have also explored predictors of clinically important gastrointestinal bleeding. In a post hoc analysis of the SUP-ICU cohort, Granholm et al. identified predictors of illness severity, such as need for circulatory support, and renal replacement therapy as important predictors of bleeding risk [18].

Critically ill patients often present with multiple concomitant physiological disturbances, including hemodynamic instability, systemic inflammation, organ dysfunction, and medication exposures, which may interact and collectively influence bleeding risk. Despite this complexity, the available data refers to individual risk factors rather than multivariable risk stratification.

A conceptual framework summarizing major physiological domains associated with bleeding risk in critically ill patients and their representation in contemporary trials is presented in Table 1.

Evidence

Early observational studies established the clinical rationale for stress ulcer prophylaxis in critically ill patients. Cook et al., in a prospective multicenter cohort study in 1994, identified respiratory failure requiring mechanical ventilation for more than 48 hours and coagulopathy as two independent predictors of clinically important gastrointestinal bleeding. These findings influenced the next ICU protocols [4].

In parallel, there are growing concerns about the use of acid suppressors and possible association with undesirable side effects such as nosocomial infections in the ICU population [5].

Over the past decade, large multicenter randomized controlled trials have re-examined acid-suppressive therapy across international ICUs. These studies reflect contemporary critical care practice and clarify its impact on clinically important outcomes, including gastrointestinal bleeding, mortality, and potential adverse events such as nosocomial infections and myocardial ischemia.

The SUP-ICU trial (2018), conducted across six European countries, enrolled critically ill adults considered at increased risk of gastrointestinal bleeding. High-risk status required the presence of at least one predefined criterion, including shock, coagulopathy, chronic liver disease, anticoagulant use, renal replacement therapy, or mechanical ventilation for more than 24 hours. Patients were randomized to receive pantoprazole 40 mg daily or placebo. Although pantoprazole reduced clinically important gastrointestinal bleeding compared with placebo (ARR: 1.7%, RR: 0.58; NNT: 59), no difference in mortality was observed (RR: 1.02, 95% CI: 0.91-1.13) [12].

Following the SUP-ICU trial, secondary analysis explored outcomes in different subgroups. A post hoc analysis by Marker et al. evaluated patients with greater illness severity and similarly found no mortality benefit despite modest reductions in bleeding risk, demonstrating the variability of risk profiles among critically ill patients [19].

Subsequent trials further explored stress ulcer prophylaxis in contemporary ICU populations, with important differences in study design and inclusion criteria.

The PEPTIC trial (2020) adopted a cluster randomized crossover design across multiple countries. Inclusion criteria were mechanically ventilated adult patients, and the study compared proton pump inhibitors and histamine-2 receptor antagonists rather than a placebo. PPIs were associated with a reduction in clinically important bleeding (RR: 0.73, 95% CI: 0.57-0.92), but there was no statistically significant difference in mortality [20].

More recently, a large, well-powered RCT, known as REVISE (Re-Evaluating the Inhibition of Stress Erosions Trial), similarly focused on invasively mechanically ventilated adult patients and compared pantoprazole with placebo in a blinded, multicenter design. This study showed good separation and definition between the groups, which were considered the primary outcome (with 85% power to detect a 1.5% difference in the primary outcome). Pantoprazole reduced clinically important bleeding (ARR: 2.5%, 95% CI: 1.6-3.3), yet no mortality benefit was demonstrated (RR: 0.94, 95% CI: 0.85-1.04) [9].

Differences in study design and inclusion criteria influence the interpretation of these trials. Large multicenter randomized studies such as SUP-ICU and REVISE demonstrate strong internal validity and provide robust estimates of treatment effects. Their external validity may be influenced by the specific eligibility criteria used to define high-risk populations, and extrapolation to the broader ICU population should be made cautiously. In contrast, the PEPTIC trial used a pragmatic cluster randomized crossover design and demonstrated greater generalizability by including broader ICU populations [9,12,20].

Other potential risk factors for gastrointestinal bleeding include acute kidney injury (AKI) and renal replacement therapy (RRT) due to uremia-induced platelet dysfunction, coagulation alterations, and frequent anticoagulation exposure. Therefore, a prespecified renal subgroup analysis of the SUP-ICU trial (SIREN) evaluated patients receiving renal replacement therapy (RRT). Bleeding incidence was numerically greater among patients requiring RRT; however, pantoprazole did not significantly reduce clinically important bleeding (OR: 0.53, 95% CI: 0.18-1.59, p>0.05), mortality, or infectious complications compared with placebo within this subgroup. The wide confidence intervals reflect limited statistical power, highlighting uncertainty in patients with renal dysfunction [17].

Additionally, other randomized clinical trials have evaluated stress ulcer prophylaxis in mechanically ventilated populations with particular attention to infectious complications such as ventilator-associated pneumonia. While modest reductions in clinically important bleeding were observed, these trials were generally underpowered to detect differences in mortality and did not demonstrate consistent increases in pneumonia or other infections. The limited sample sizes and subgroup-specific designs restrict the application to a broader patient population [21].

At the same time, contemporary studies have evaluated stress ulcer prophylaxis in mechanically ventilated patients, specifically examining the influence of early enteral nutrition, an intervention that may reduce mucosal vulnerability. Across these trials, the incidence of clinically important gastrointestinal bleeding was consistently low, and no significant additional benefit of proton pump inhibitors over placebo was observed. In patients tolerating early enteral feeding, bleeding rates remained below 2%-3%, with no differences in mortality or infectious complications. These findings suggest that early enteral nutrition may protect the gastric mucosa, potentially reducing the need for pharmacologic prophylaxis in critically ill patients. Subsequent post hoc analyses by the author Borthwick et al. supported this association [22-26].

For this reason, reassessment of prophylaxis during the ICU course is important. As patients stabilize and risk factors resolve, daily evaluation and timely discontinuation of acid-suppressive therapy may help reduce unnecessary exposure. Inappropriate continuation of stress ulcer prophylaxis after hospital discharge has been reported, emphasizing the importance of deprescribing when the initial indication is no longer present [13,27].

Meta-analyses have synthesized these findings, demonstrating a reduction in clinically important gastrointestinal bleeding with acid-suppressive therapy, with no significant mortality benefit, and reinforced that substantial clinical heterogeneity exists across included trials, particularly regarding definitions of high-risk populations, baseline bleeding incidence, and concurrent use of early enteral nutrition [28-30].

Contemporary guidelines reflect this evolution. The SCCM/ASHP guideline recommends risk-stratified prophylaxis in high-risk critically ill patients while advising against routine universal prophylaxis; however, the certainty of evidence remains limited. The recommendations are conditional and acknowledge the variability in risk stratification in modern ICU populations [13].

**Figure 2:**
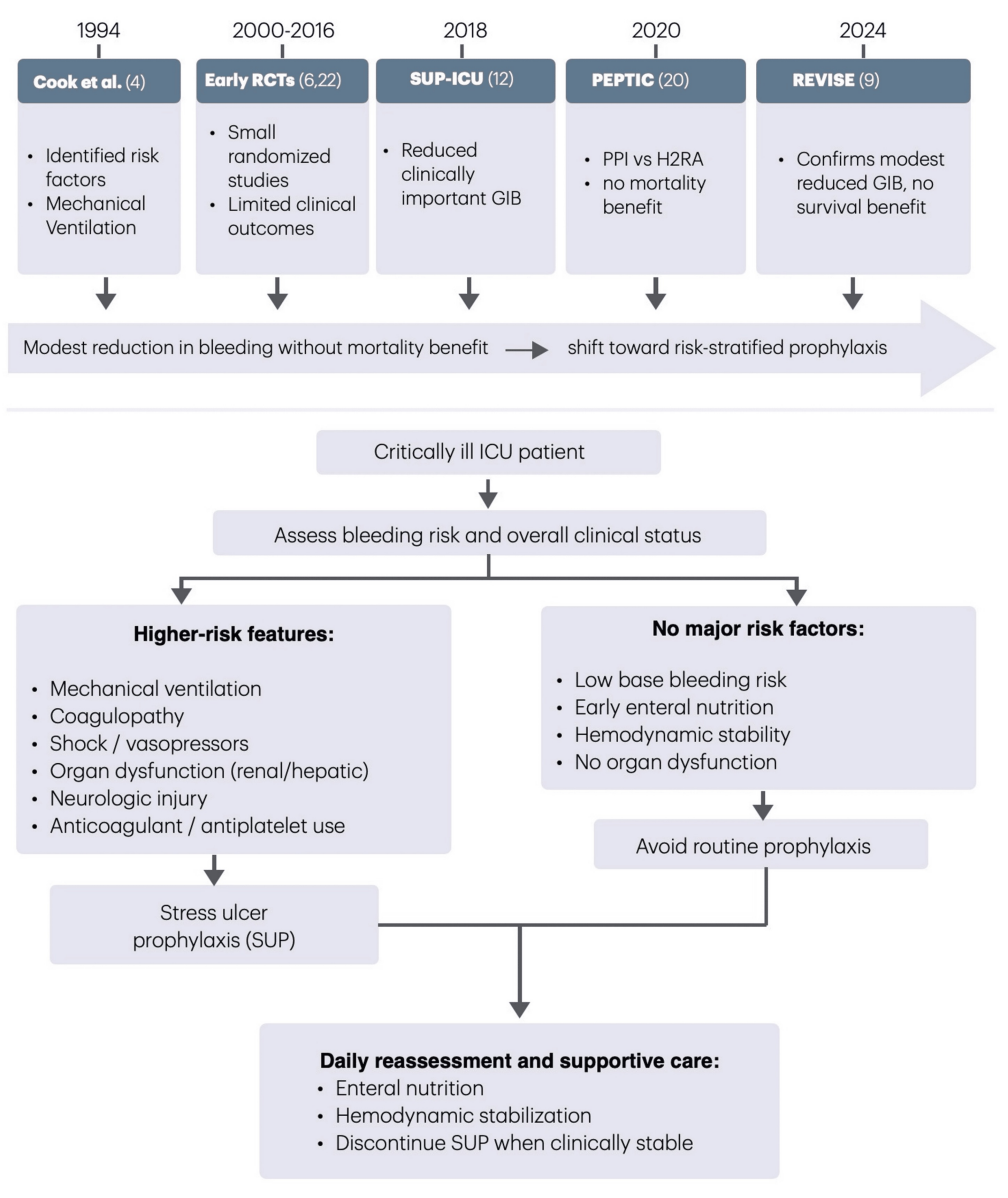
Evolution of evidence and clinical approach to stress ulcer prophylaxis in critical care This figure summarizes key milestones from early observational studies to contemporary randomized controlled trials, highlighting the consistent finding of a modest reduction in clinically important gastrointestinal bleeding without mortality benefit. The lower panel illustrates a practical risk-based approach to guide selective use of stress ulcer prophylaxis in modern ICU practice. ICU: intensive care unit, RCT: randomized controlled trial, PPI: proton pump inhibitor, SUP: stress ulcer prophylaxis, H2RA: histamine-2 receptor antagonist, GIB: gastrointestinal bleeding

Table 2 summarizes the studies on stress ulcer prophylaxis in critically ill adults. The evolution of evidence supporting a shift toward risk-stratified stress ulcer prophylaxis in critically ill patients is summarized in Figure 2.

**Table 2:** Major studies on stress ulcer prophylaxis in critically ill adults Summary of key RCTs and foundational studies informing current clinical practice RCT: randomized controlled trial, ICU: intensive care unit, IV: intravenous, EN: enteral nutrition, GI: gastrointestinal, PPI: proton pump inhibitor

Study	Year	Sample size (number)	Study design	Population	Intervention/comparator	Key findings
Cook et al. [4]	1994	2,252	Prospective cohort	Critically ill ICU patients	Observational risk factor analysis	Prolonged mechanical ventilation (>48 hours) and coagulopathy identified as major independent predictors of clinically important GI bleeding.
Brophy et al. [6]	2010	185	RCT	Neurosurgical ICU patients	Lansoprazole versus IV famotidine	Both therapies increased gastric pH; clinically significant bleeding events were uncommon.
Selvanderan et al. (POP-UP) [22]	2016	214	Randomized controlled exploratory trial	Critically ill ICU patients	Pantoprazole versus placebo	Low incidence of clinically important bleeding in contemporary ICU populations.
El-Kersh et al. [23]	2018	102	RCT	Critically ill patients receiving enteral nutrition	Enteral nutrition alone versus pharmacologic prophylaxis	No additional reduction in bleeding with acid suppression when enteral nutrition was provided.
Nourian et al. [24]	2018	50	RCT	Critically ill ICU patients	Enteral nutrition versus EN + ranitidine	Similar bleeding incidence between groups, suggesting a potential protective role of enteral feeding.
Krag et al. (SUP-ICU) [12]	2018	3,298	Multicenter RCT	ICU patients at risk of GI bleeding	Pantoprazole versus placebo	Pantoprazole reduced clinically important bleeding but showed no mortality benefit.
Schefold et al. (SIREN subgroup) [17]	2019	568	Prespecified subgroup analysis of RCT	ICU patients receiving renal replacement therapy	Pantoprazole versus placebo	No significant reduction in bleeding or mortality in patients receiving dialysis.
Young et al. (PEPTIC) [20]	2020	26,982	Cluster randomized crossover trial	Mechanically ventilated ICU patients	PPI versus H2-receptor antagonist	PPIs reduced clinically important bleeding but did not improve mortality.
Granholm et al. [18]	2021	3,291	Post hoc analysis of RCT	ICU patients at risk of bleeding	Risk factor analysis	Illness severity, circulatory support, and renal replacement therapy were the strongest predictors of bleeding risk.
Cook et al. (REVISE) [9]	2024	4,800	Multicenter RCT	Mechanically ventilated ICU patients	Pantoprazole versus placebo	Pantoprazole modestly reduced bleeding but showed no survival benefit.

Discussion

Stress ulcer prophylaxis is widely used in critical care to prevent stress-related mucosal damage (SRMD) and its complications. Evidence from randomized trials suggests that proton pump inhibitors reduce the incidence of clinically important gastrointestinal bleeding in patients considered at higher risk, although a consistent mortality benefit has not been demonstrated. Methodological differences across trials should be considered when interpreting these results, as cluster randomized crossover designs, for example, may attenuate group differences and potentially bias results toward the null hypothesis. Within this context, several studies in recent years have evaluated prophylaxis for SRMD, but relatively few have focused specifically on critically ill populations, and the available studies demonstrate important heterogeneity [9,12,13,20].

The central observation from this review is that part of this variability derives from differences in how bleeding risk has been defined across trials. Early observational studies identified prolonged mechanical ventilation and coagulopathy as major independent predictors of clinically important bleeding. However, subsequent randomized trials have evaluated bleeding risk differently. Variations in inclusion criteria and study populations may lead to inconsistent findings and limit the precision of current recommendations, complicating the interpretation of available evidence and contributing to ongoing uncertainty [3,18].

Additionally, modern ICU practice has evolved, with improvements such as early volume resuscitation, improved hemodynamic stabilization, earlier initiation of enteral nutrition, and overall advances in critical care. These changes may have lowered the baseline bleeding incidence compared with earlier decades and contributed to the modest absolute treatment effects observed in large, randomized trials such as SUP-ICU and REVISE [2,5,9,18,22-24,26].

Secondary analyses, such as SUP-ICU, RENAL subgroup, evaluations of illness severity (represented by higher Sequential Organ Failure Assessment (SOFA) and Simplified Acute Physiology Score II (SAPS II) scores), and the need for circulatory support and renal replacement therapy, suggest that bleeding risk in critically ill patients is associated with overall disease severity and multiple predictors rather than isolated variables [18].

As a result, the current clinical challenge is determining which patients should be considered “high risk” and when to prescribe stress ulcer prophylaxis without unnecessarily exposing patients to treatments that may not provide meaningful benefit and even lead to a nosocomial infection. The possibility of unintended harm reinforces the importance of careful patient selection.

High-quality outcome data remain limited for specific subgroups of critically ill patients, including those with neurologic injury, renal replacement therapy, or complex comorbid conditions. Variability in population selection complicates extrapolating pooled results to the broader ICU population and highlights the limitations of approaches based on single risk factors [7,17,18].

The widespread implementation of early enteral nutrition represents another important modifier of baseline bleeding risk that was not consistently present in earlier ICU scenarios. Improvements in supportive care may reduce mucosal vulnerability, thereby lowering the absolute benefit of routine prophylaxis in some patients [22-26].

Current clinical practice guidelines reflect this uncertainty. Most recommend stress ulcer prophylaxis in patients with established major risk factors while advising against routine use in low-risk individuals. Consequently, clinical decision-making remains dependent on clinician judgment and local practice patterns rather than on precise risk stratification tools [13,29].

Future research should aim to develop risk assessment models that integrate multiple clinical variables, such as mechanical ventilation, coagulopathy, shock, vasopressor use, renal failure, chronic liver disease, neurologic injury, major surgery or trauma, sepsis or systemic inflammation, and enteral nutrition tolerance, rather than relying on isolated risk factors. Prospective studies reflecting contemporary ICU populations are needed to better identify patients most likely to benefit from prophylaxis and to support more individualized, evidence-informed practice.

Clinical implications for practice

Current evidence suggests that stress ulcer prophylaxis should not be administered universally to all critically ill patients. Instead, a risk-stratified approach is recommended. In clinical practice, risk stratification is based on the presence of major and additional risk factors that increase the likelihood of clinically important gastrointestinal bleeding. Patients with established major risk factors, including prolonged mechanical ventilation, coagulopathy, shock, vasopressor use, chronic liver disease, or renal replacement therapy, appear more likely to benefit from pharmacologic prophylaxis [3,12].

Additional factors, such as higher illness severity scores (e.g., SOFA or SAPS II), sepsis, and organ dysfunction, may further modify bleeding risk and should be considered in the overall clinical assessment. In contrast, patients without established risk factors demonstrate a low baseline incidence of clinically important gastrointestinal bleeding in modern ICU practice, particularly when early enteral nutrition is provided [22-24].

Daily reassessment of prophylaxis is therefore recommended. As patients stabilize and risk factors resolve, discontinuation of acid-suppressive therapy may help minimize unnecessary exposure and reduce potential adverse effects [13,27].

Until validated multivariable risk prediction tools are available, clinical decision-making remains dependent on clinician judgment and local practice patterns.

Limitations

This review has several limitations. As a narrative review rather than a systematic review, it does not follow a prospectively registered protocol (PROSPERO) and does not include a formal risk-of-bias assessment or quantitative statistical synthesis.

To enhance transparency and reduce potential bias, a structured literature search and predefined eligibility criteria were applied. In addition, the PICO framework was used to guide the research question, and elements of the Preferred Reporting Items for Systematic Reviews and Meta-Analyses (PRISMA) were incorporated to document the study selection process.

Despite the inherent limitations of a narrative review, this study provides a structured synthesis of the available evidence, offering clinically relevant insights.

## Conclusions

Stress ulcer prophylaxis prevents clinically important gastrointestinal bleeding in high-risk ICU populations, although the absolute benefit is modest and dependent on baseline bleeding risk. In parallel, advances in critical care, including improved hemodynamic resuscitation, early enteral nutrition, and overall improvements in supportive care, have likely reduced the incidence of stress-related mucosal damage compared with decades ago and, as a result, altered the balance between benefits and potential harms of prophylactic therapy. Current guideline recommendations therefore support a targeted approach, favoring prophylaxis in patients with established major risk factors rather than routine universal administration. However, the absence of multivariable prediction models limits the precision with which clinicians can identify the patients most likely to benefit. Therefore, developing evidence-based guidance incorporating risk prediction is the next step toward more individualized prophylaxis. Continued research aimed at improving bleeding risk prediction and defining the optimal duration of therapy will be essential to further refine clinical practice.
